# Adolescents show collective intelligence which can be driven by a geometric mean rule of thumb

**DOI:** 10.1371/journal.pone.0204462

**Published:** 2018-09-24

**Authors:** Christos C. Ioannou, Gabriel Madirolas, Faith S. Brammer, Hannah A. Rapley, Gonzalo G. de Polavieja

**Affiliations:** 1 School of Biological Sciences, University of Bristol, Bristol, United Kingdom; 2 Instituto Cajal, Consejo Superior de Investigaciones Científicas, Madrid, Spain; 3 Champalimaud Research, Champalimaud Center for the Unknown, Lisbon, Portugal; 4 Department of Psychology, University of Bath, Bath, United Kingdom; University of Massachusetts, UNITED STATES

## Abstract

How effective groups are in making decisions is a long-standing question in studying human and animal behaviour. Despite the limited social and cognitive abilities of younger people, skills which are often required for collective intelligence, studies of group performance have been limited to adults. Using a simple task of estimating the number of sweets in jars, we show in two experiments that adolescents at least as young as 11 years old improve their estimation accuracy after a period of group discussion, demonstrating collective intelligence. Although this effect was robust to the overall distribution of initial estimates, when the task generated positively skewed estimates, the geometric mean of initial estimates gave the best fit to the data compared to other tested aggregation rules. A geometric mean heuristic in consensus decision making is also likely to apply to adults, as it provides a robust and well-performing rule for aggregating different opinions. The geometric mean rule is likely to be based on an intuitive logarithmic-like number representation, and our study suggests that this mental number scaling may be beneficial in collective decisions.

## Introduction

Making decisions in groups can greatly improve cognitive performance [1]. This effect is of widespread interest in psychology, management and political science, partly due to the importance of social interactions in society from small everyday decisions to governmental panels deciding issues of policy [2]. Studies in this area of research range from exploring optimal methods to statistically aggregate large samples of estimates [3], to how individuals use information from others [4], to decision making in groups of freely interacting individuals [5]. Despite this extensive research, our understanding is however incomplete as this previous work has been limited to studies on adults; little is known about how collective intelligence develops during the approach to adulthood.

Complementing research on adult humans, collective intelligence has been documented in a range of non-human animals [6]. Often, the emphasis in studies of non-humans is how cognitively-limited individual units (e.g. cells, ants, fish or robots) exchange and process information to improve decision making by the group. Such an approach can also be applied to humans: the less developed cognitive and social skills [7–9] of younger people may limit their ability to make decisions collectively compared to adults, forming an intriguing bridge between research in adults and non-human animals. Previous studies in adults have shown the importance of information exchange and social sensitivity in collective accuracy [10,11], and it has been argued that behaviour related to social interactions is still under development until as late as 14–15 years of age [12]. For example, when opinions differ and a single group consensus needs to be reached, compromise is required, and this may not be as developed in younger people [13]. Similarly, aggregating multiple opinions to improve accuracy (often referred to as the ‘wisdom of crowds’ [4]) relies on appropriate numerical processing to aggregate the opinions, for example by taking the median value. As people develop through adolescence, both the social and cognitive skills which are required for effective collective intelligence should improve, and hence collective decisions should become more accurate.

Research with younger people has been carried out on group work in classroom settings although this has, understandably, been aimed at improving individual learning and behaviour [14,15] rather than investigating the performance of collective decisions relative to those made by individuals. Our study was designed to test whether the improved performance often observed in groups versus single individuals is seen in younger people, if this effect changes with age, and how initial individual estimates are aggregated by the group during collective decision making. We performed two experiments using a simple numerical estimation task [3,16]. In the first, a range of ages were tested and participants were asked for an individual (i.e. independent) initial pre-discussion estimate, a consensus group estimate after a period of unconstrained discussion in groups, and a post-discussion individual estimate that could again vary between individuals within each group. Comparing the degree of error (the estimate minus the correct number of sweets) across these stages allowed us to determine whether collective intelligence was evident, and whether this varied with the age of the participants. Further analysis explored how the three initial estimates were processed to give the group estimate, whether and how this varied between groups, and the consequences of using different ‘rules’ to process the initial individual estimates in terms of minimising errors. This task generated a positively (right) skewed distribution of initial estimates, which is likely to favour particular rules in determining the group estimate. In Experiment 2, we modified the task to reduce the extent of outliers, testing whether collective intelligence in adolescents is generalizable to less skewed distributions of initial estimates. Participants were asked for initial, independent estimates and then a consensus group estimate after a period of unconstrained discussion in groups.

Participants as young as 11 years old (S1 Fig) were asked to guess the number of sweets in jars (S2 Fig). We used a simple task of estimating the number of sweets in jars so that the young participants would feel engaged with the task [17] and would not assume they are better or worse at the task compared to other participants, which may occur with knowledge-based questions. A repeated measures design was used in both experiments, where each group of 3 participants was asked to give an individual initial estimate that was independent from their other group members, a group consensus estimate, and, in Experiment 1 only, another individual estimate post-discussion. Repeatedly testing the same groups, rather than assigning individuals to ‘individual’ or ‘group’ treatments allowed us to analyse changes in accuracy, and track how the group consensus was made, based on the initial estimates in each group. Such repeated measures designs are common in group performance research [4]. A group size of 3 was used as group work in schools is most often conducted in small groups, previous group research with children has used this group size [18,19], social loafing is more likely in larger groups [20] and undergraduate participants preferentially form groups of this size in classroom tasks [21]. A smaller group size also made analysis of how initial estimates were collectively processed more tractable.

## Results

### Experiment 1

**Fig 1:**
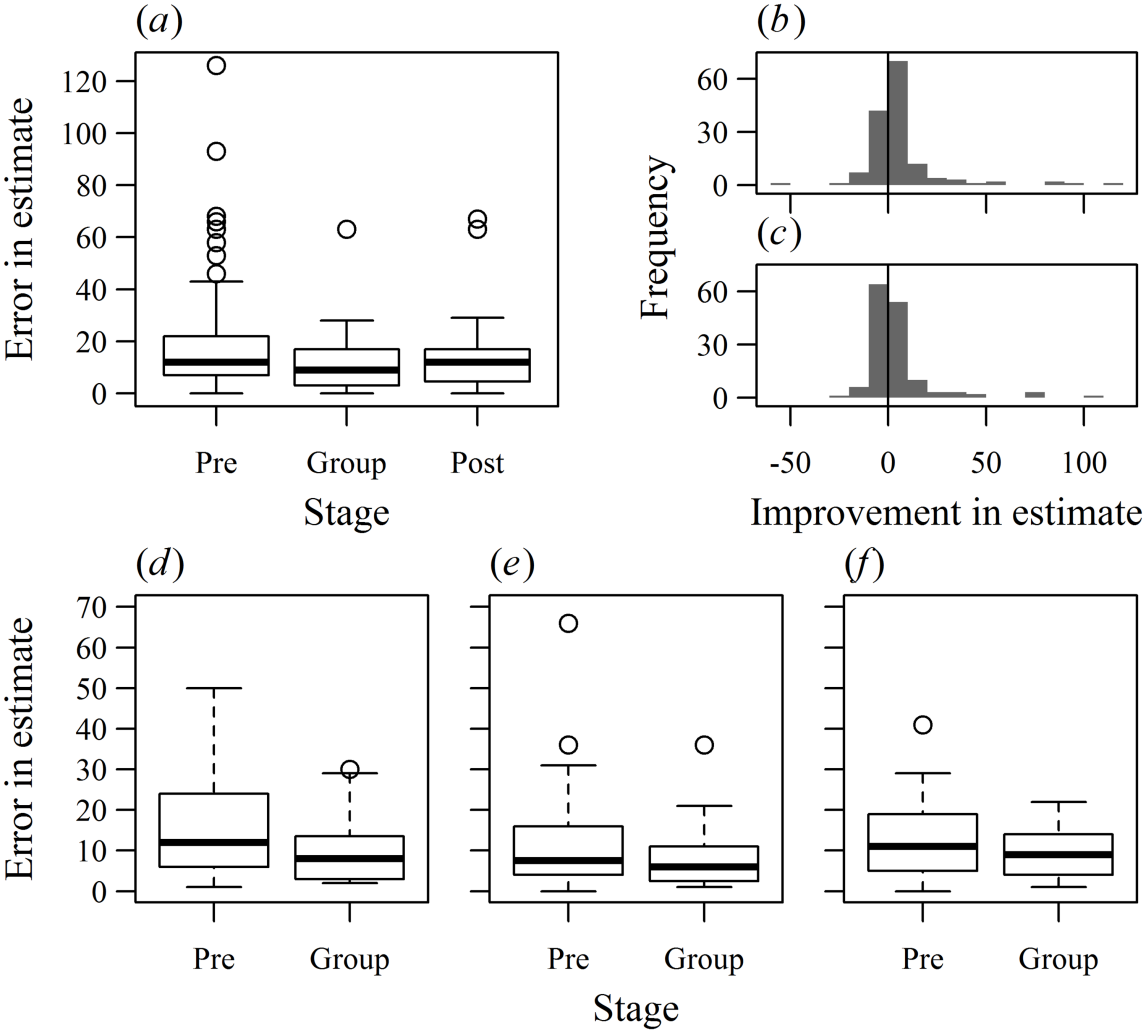
Collective intelligence in adolescents. In experiment 1 (a,b,c), the errors in the pre-discussion initial estimates are significantly greater than both the group consensus (neg. bin. GLMM: z = 6.74, p < 0.001) and post-discussion estimates (z = 5.27, p < 0.001). Group estimates tended to have less error on average than individual estimates given after discussion although this effect was not statistically significant (z = -1.77, p = 0.077). The frequency distributions of the error in the initial estimates minus the error in the group consensus estimates (b) or post-discussion individual estimates (c) per participant show that while most participants only gain a small improvement in reduced errors (b) or show little change in errors (c), a minority of individuals vastly reduce the error in their estimates after group discussion. In Experiment 2, the absolute error in the consensus group estimate was lower than the initial (pre) estimates in all three treatments (proportion of black sweets: d: 48/200, e: 94/190, f: 121/160). The box plots show the median (thick black lines), interquartile range (enclosed by the boxes), 1.5 × the interquartile range beyond the boxes (whiskers) and outliers beyond the whiskers (open circles).

There was a strong positive correlation between individual estimates within groups after, but not before, group discussion (S1 Fig). This convergence of estimates within groups demonstrates social influence after information is shared during the group discussion stage [4]. A collective intelligence effect was found with an overall reduction in errors after group discussion (Fig 1a, negative binomial Generalised Linear Mixed Model (neg. bin. GLMM): LRT_2,433_ = 50.66, p < 0.001), an effect which was independent of the participants’ gender or age (S1 Table). The majority of participants improved their estimate when giving their group consensus estimate compared to their initial estimate (96 participants reduced errors, while errors did not change or increased for 51 participants; Fig 1b). However, when comparing errors in the initial and post-discussion individual estimates, when estimates within groups could again vary, the probability that participants improved their estimate was close to 0.5 (76 improved, 71 did not; Fig 1c). This difference between the group consensus and post-discussion stages was statistically significant (binomial GLMM: LRT_1,288_ = 13.49, p < 0.001), suggesting that the group consensus stage was the most beneficial in reducing errors. Whether estimates improved after group discussion was independent of the participants’ ages (S1 Table), although female participants were more likely to improve their estimate after group discussion than males (LRT_1,288_ = 8.02, p = 0.0046).

**Fig 2:**
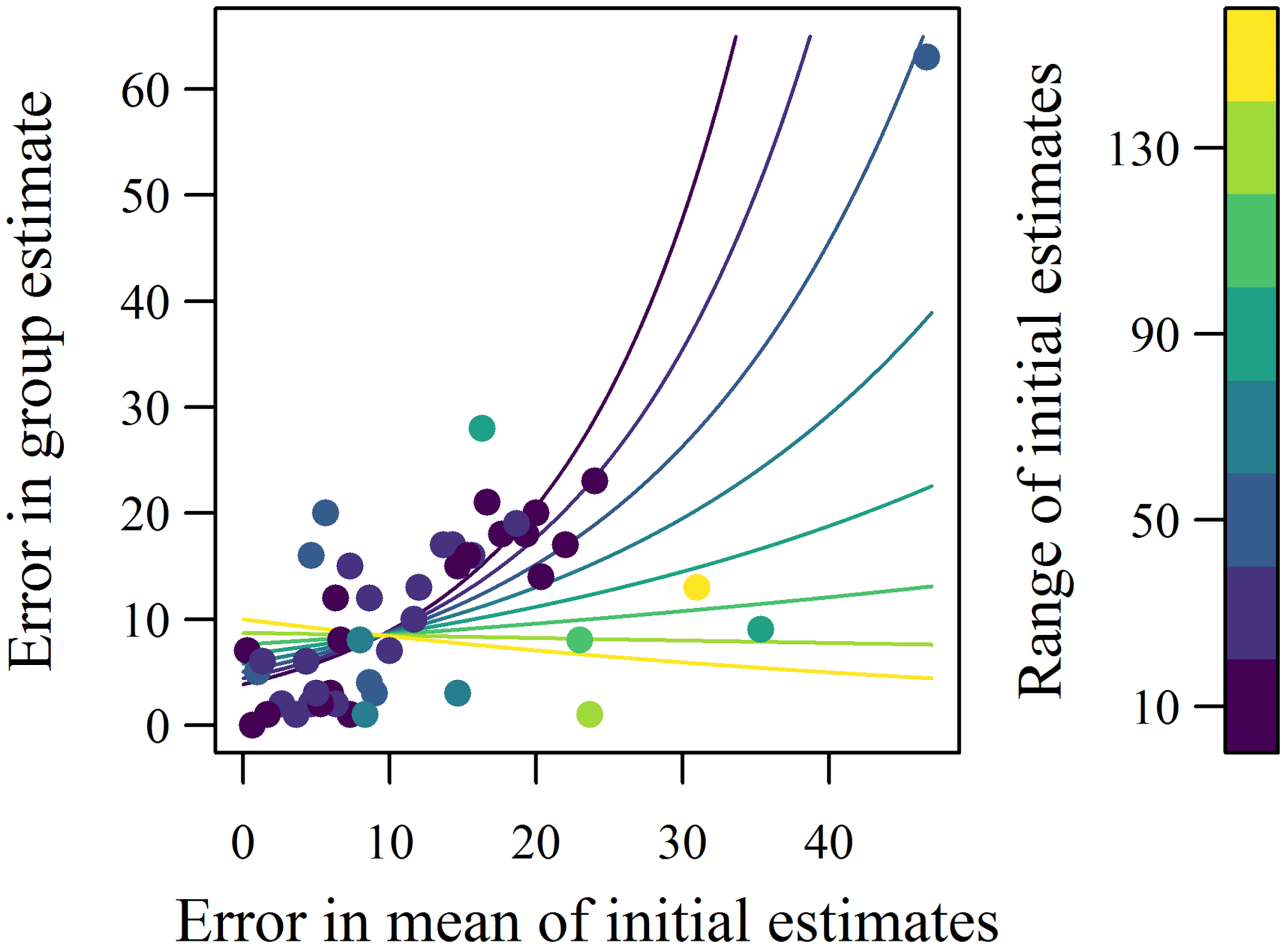
The effect of disagreement in initial individual estimates on improving group estimates in Experiment 1. Disagreement is measured as the range of initial estimates in each group. The colours represent this range, binned every twenty units. Coloured lines are fits for each range interval, calculated from the GLM coefficients which includes the significant interaction term between the error of the (arithmetic) mean and the range of the initial estimates. The main effects of gender and mean age are included in the calculation of fitted values, each fixed at their mean values in the data. If the error in the mean initial estimates directly determines the error in the group estimate, there should be a positive linear relationship between the two variables (as occurs with the darker points, i.e. groups with a smaller range of initial estimates). No relationship between the two errors (mean initial and group) can occur if the group discussion revises the estimate enough that the error of the mean initial estimates is no longer predictive of the error of the group estimate, as occurs with the more lightly coloured points.

**Fig 3:**
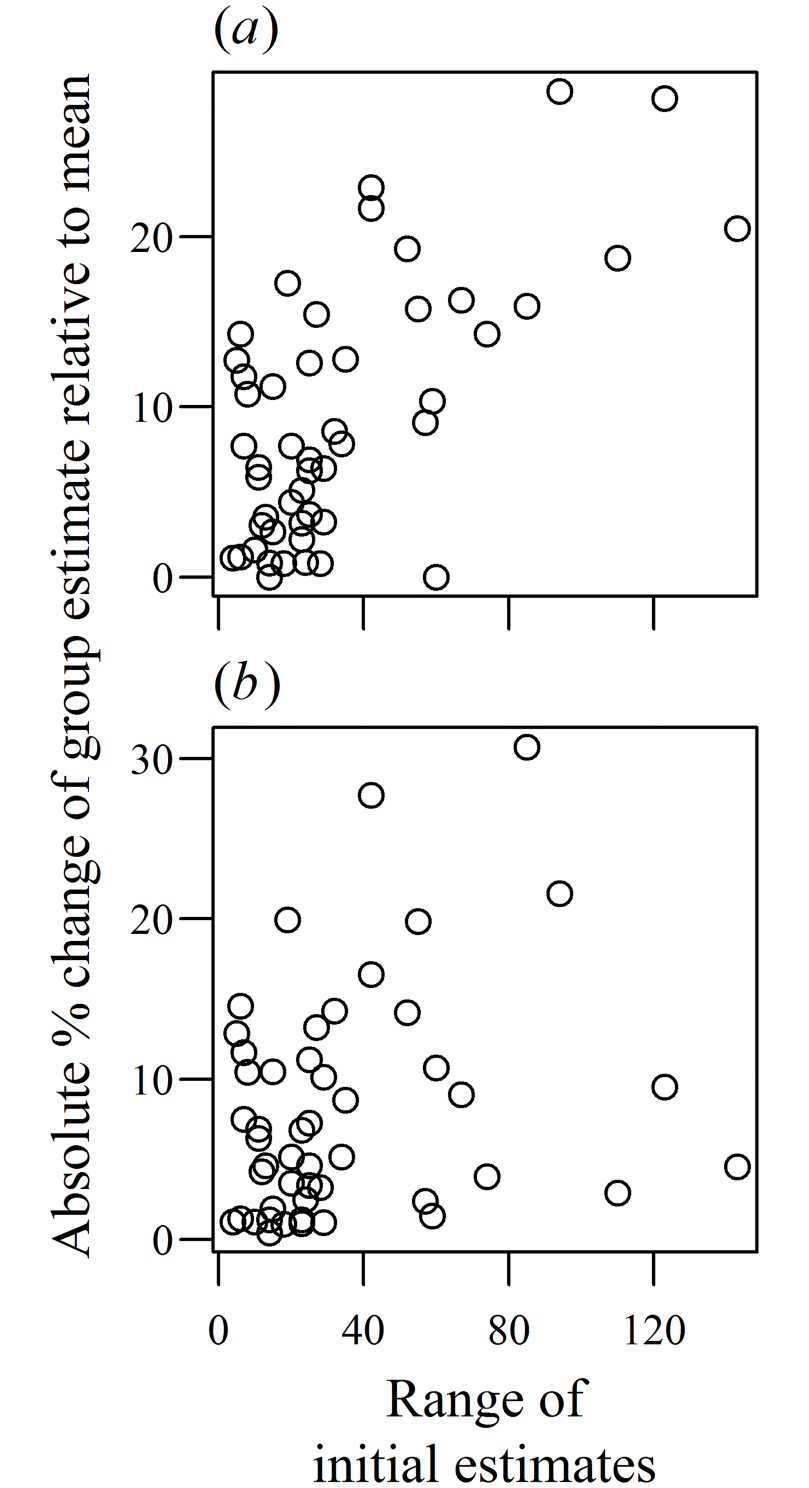
How initial disagreement shifts the consensus group estimates away from the mean of initial estimates in Experiment 1. Disagreement is the range of initial estimates. (a) shows the absolute percentage difference relative to the arithmetic mean, and (b) relative to the geometric mean.

To explore how accuracy was affected by the disagreement of initial estimates within each group, the effect of the range of initial individual estimates on the error in the consensus group estimate was analysed (Fig 2). When the error of the group estimate was expected to be high due to the mean of the initial estimates being inaccurate, more disagreement in initial estimates resulted in better group estimates than expected (neg. bin. GLM: LRT_1,43_ = 4.44, p = 0.035). In other words, higher disagreement lead to greater accuracy, weakening the relationship in error between initial and group estimates. This reflects a similar trend found in adults [22], and we also find that groups with a greater range in initial estimates shifted more from the mean of their estimates when giving their group estimate (Fig 3a, neg. bin. GLM: LRT_1,45_ = 18.31, p < 0.001).

The wide range of initial estimates in some groups was usually due to a single estimate, i.e. an outlier, which differed from the estimates given by the other two group members. This was evident in the lack of an effect the range had on the mean of the two initial estimates that were closest to one another, particularly when compared to the positive relationship between the range and the mean of all initial estimates (S3 Fig). The groups’ consensus estimates were, however, intermediate between the two, having a significantly steeper relationship with the range than the mean of the two estimates that were closest to one another (neg. bin. GLMM: z = -3.54, p < 0.001), but a relationship less steep than expected from the mean of all initial estimates (z = 2.23, p = 0.026). This shows that the weight given to outlier estimates was reduced compared to simple arithmetic averaging of all initial estimates but not dismissed entirely in the group consensus decision.

**Fig 4:**
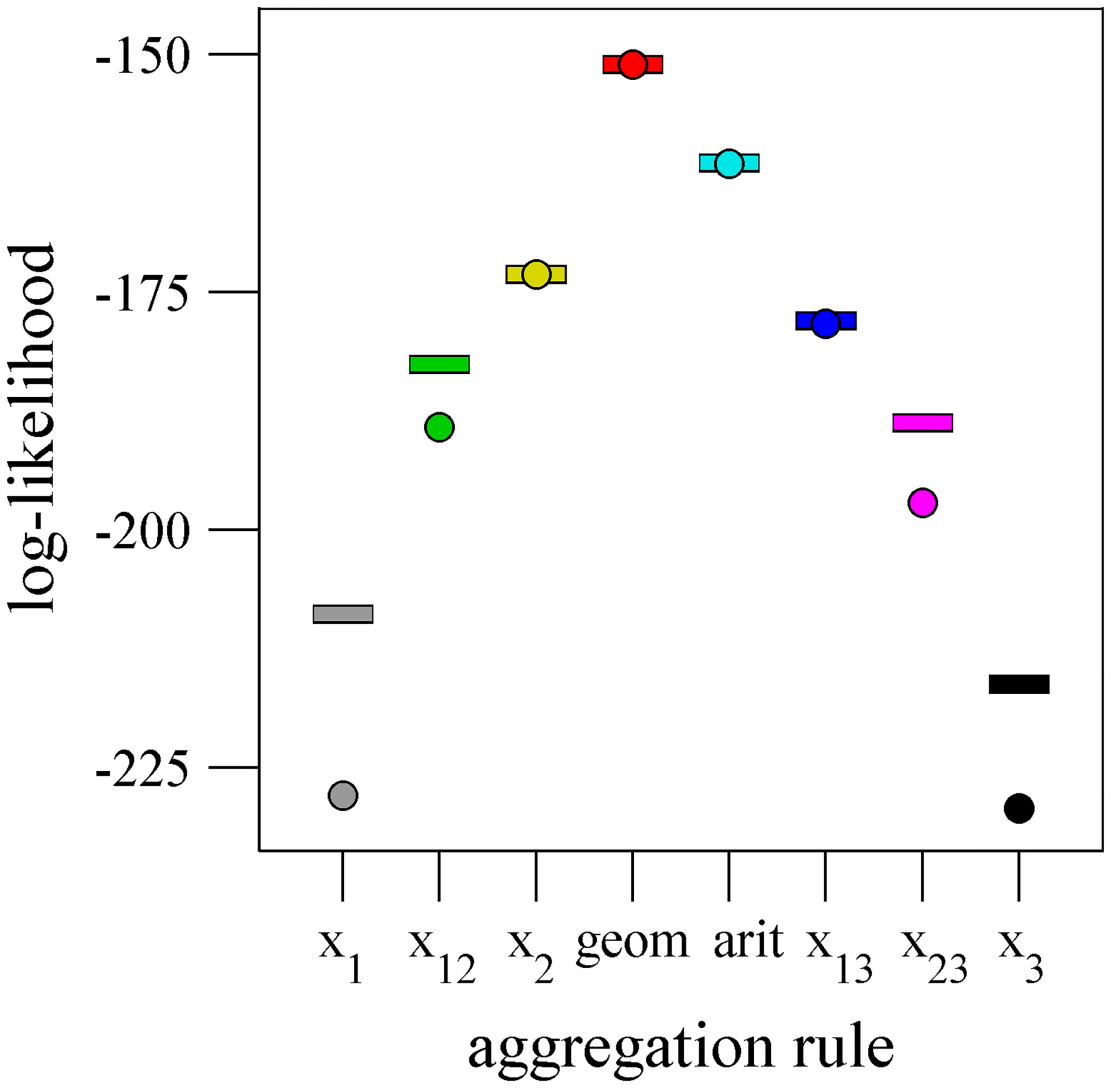
Fits of different aggregation rules to the observed group consensus estimates in Experiment 1. Observed log-likelihoods of eight different rules for aggregating initial estimates (circles) are plotted with the log-likelihoods when the noise (dashes) added to each estimation maximizes the log-likelihood (S4 Fig, S1 Methods). For the median (x_2_), geometric (geom) and arithmetic (arit) means, and mean of the lowest and highest estimates in each group (x_13_), the fits to the data are close to maximal. The other rules tested are: the lowest estimate (x_1_), mean of the lowest and median estimate (x_12_), mean of median and highest estimate (x_23_), and highest estimate (x_3_). The strategies are sorted in the x axis in an order that results in increasing values for many of the groups.

Given the right-skewed distribution of the initial estimates (S1 Fig), a potential mechanism to reduce the effect of outliers within groups would be to use the geometric, rather than arithmetic, mean. It has been demonstrated recently that adults integrate estimates from others using a rule that approximates the geometric mean [23], although it is unknown whether groups (including groups of adults) reaching a consensus will also use this rule. When comparing models of the group consensus estimates as noisy estimates of various cognitively simple aggregation rules (Fig 4, S1 Methods), using the geometric mean to aggregate initial estimates gave the best fit to the data (Fig 4, S4 Fig). Furthermore, despite the strong effect disagreement had on how much groups shifted from the arithmetic mean (Fig 3a), disagreement in initial estimates was only marginally, and not statistically significantly, related to the shift from the geometric mean (Fig 3b, neg. bin. GLM: LRT_1,45_ = 3.47, p = 0.062). The close match between the groups’ estimates and the geometric mean of the initial estimates is particularly evident when both are plotted against the range of initial estimates (S4 Fig, neg. bin. GLMM: z = 0.18, p = 0.86).

Estimates given by individuals before and after group discussion, when individuals were again free to deviate from the group consensus, were compared to further explore how initial estimates in groups were aggregated. Given the data [24], a model with the log10 initial estimates as an explanatory variable predicting the post-discussion individual estimates (S1 Table) was more likely than one where the initial estimates were untransformed (S5 Fig: neg. bin. GLMM ΔAICc log10 model = 0.0, ΔAICc untransformed model = 9.6). The group interaction thus has a logarithmic-like effect on individual estimates, an effect consistent with an approximate geometric mean aggregation rule, as the logarithm of the geometric mean is the arithmetic mean of the logarithms.

**Fig 5:**
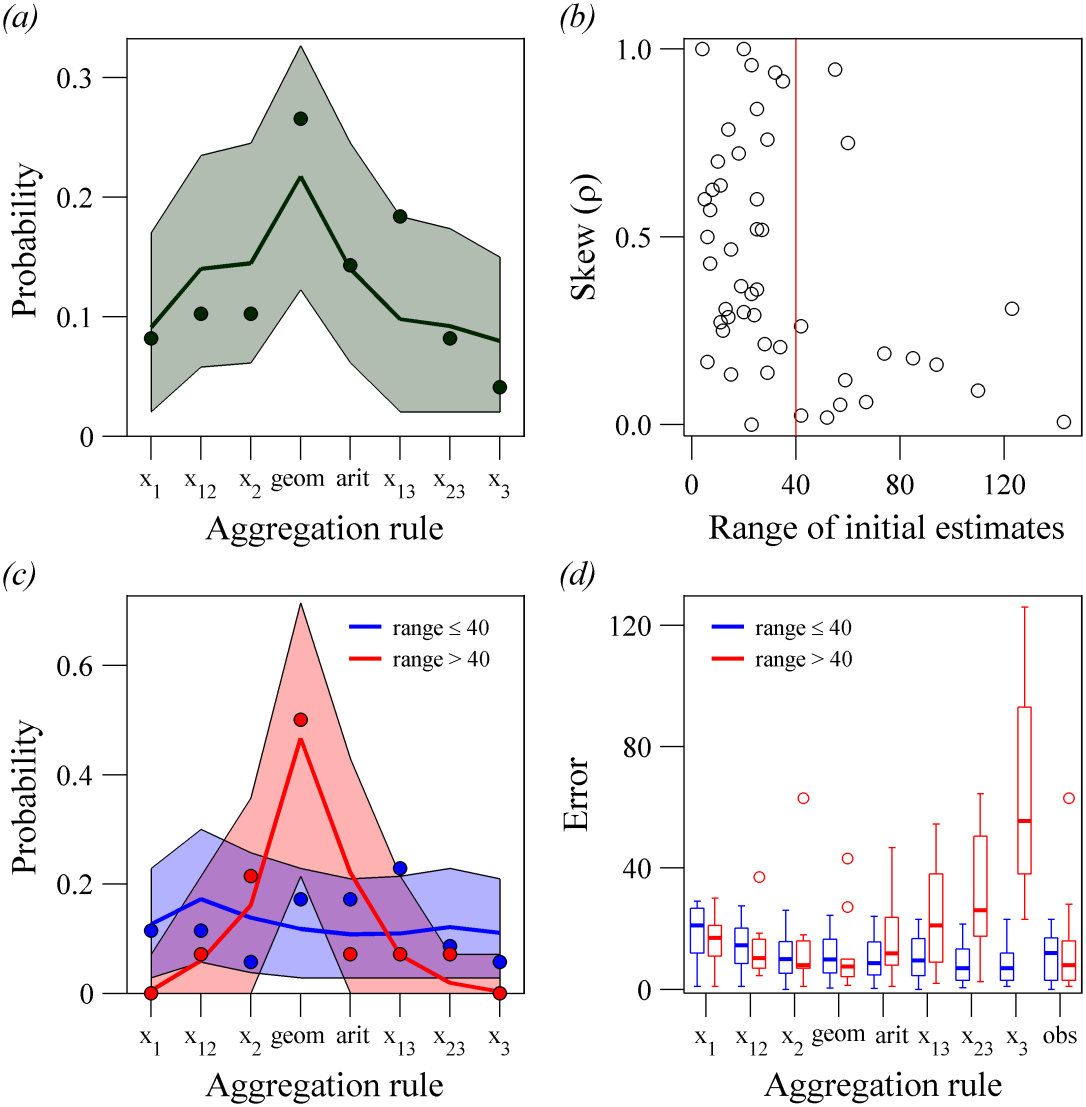
The use and consequences of different aggregation rules in Experiment 1. (a) Probability of each aggregation rule being the closest to the observed consensus estimates of the groups (filled circles). Also plotted is the probability (mean is black line, and shaded region is 95% confidence intervals) that the aggregation rule is the closest to the observed consensus estimates when a ‘noisy’ geometric mean simulation is instead used to aggregate the initial estimates (S1 Methods). (b) Range and skew in initial estimates for each group. The range of initial estimates is plotted against the relative distribution of the estimates. Skew is *ρ* = (*x*_2_−*x*_1_)/(*x*_3_−*x*_1_), and is close to zero if the highest estimate (*x*_3_) is a relative outlier, and close to one if lower estimate (*x*_1_) is a relative outlier. The threshold between groups of low and high range (red line) is an approximate point that separates the region with any configuration (≤40) to the region with the two lower estimates being much closer to each other than to the higher (>40). (c) As (a), but separately for groups with a low range of estimates (blue dots, line and shaded area) and high range of estimates (red dots, line and shaded area). (d) Absolute error if each of the strategies had been followed exactly by groups with low range (blue) and high range (red). The notation for strategies as in Fig 4, with a final column added in (d) for the error of the observed group consensus estimates. The threshold used to define groups with low and high ranges did not have any effect on the trends in (c) and (d) (S6 Fig). The box plots show the median (thick black lines), interquartile range (enclosed by the boxes), 1.5 × the interquartile range beyond the boxes (whiskers) and outliers beyond the whiskers (open circles).

Despite the evidence that the geometric mean provides a good fit to the overall observed consensus estimates (Fig 4), it is feasible that different methods were used to decide collectively, especially because the level of disagreement may influence how group decisions are made [22]. We find that although the geometric rule appeared to be used most frequently, there is still room to consider the use of the other aggregation rules, with each alternative rule (Fig 5a, S1 Methods) being the closest to the group consensus estimate at least twice. The rules we compare are relatively cognitively undemanding and hence we believe they are feasible for the young participants in our study to use, whether consciously, such as selecting the initial estimate in between the smallest and largest (i.e. the median), or intuitively with a heuristic that is more likely to occur with rules such as the arithmetic mean. However, the frequency of using alternative rules, other than using the mean of the lowest and highest estimates, was within the 95% confidence intervals of assuming the geometric mean heuristic was used to aggregate initial estimates in each group, with a level of noise added that matched the noise in estimates in the experiment (Fig 5a, S1 Methods). After splitting the data into groups with a low or high range of pre-discussion initial estimates (Fig 5b), there were several potential rules being applied in groups with low range, although it is more difficult to statistically distinguish between different rules when the range is smaller as, by definition, initial estimates are more similar to one another (e.g. S6 Fig). At high ranges, the geometric mean was clearly the most common strategy (Fig 5c).

We tested the consequences of using different aggregation rules for the accuracy of group decision making. For groups with low range, only the highest estimate and the average of the highest and median estimates significantly outperformed the geometric mean rule (Fig 5d, S2 Table). In contrast, for groups with high ranges, the geometric mean outperformed all alternatives. The rules that outperformed the geometric mean at low group ranges and the rule that was used more than expected compared to the noisy geometric rule at low ranges gave particularly large errors in groups with high ranges (Fig 5d). Thus, the geometric mean provides a robust and generally high performing aggregation rule, particularly when there is disagreement in estimates within a group, and this trend matches its preferential usage (Fig 5c). There was no difference in accuracy between the geometric mean of the initial estimates and the group consensus estimates across all groups, or only in groups with low or high ranges of initial estimates (S2 Table).

### Experiment 2

As in Experiment 1, group discussion significantly reduced errors in the participants’ estimates relative to initial estimates (Fig 1d, 1e and 1f, S3 Table, neg. bin. GLMM: individual vs. group estimate: LRT_1,417_ = 13.90, p < 0.001). This effect was unaffected by the proportion of sweets in each jar (the treatment × individual vs. group estimate interaction was not significant: LRT_2,415_ = 0.82, p = 0.66), despite the different distributions of initial individual estimates in each treatment (S7 Fig). Ranges of initial estimates were not as large as in the first experiment, thus it was difficult to distinguish whether particular aggregation rules were being used. However, we still find that in groups with larger ranges, the error in the (arithmetic) mean of the initial estimates was less predictive of the error in the group consensus estimate compared to groups with low ranges in initial estimates (S8 Fig, S3 Table, neg. bin. GLMM: absolute error in mean initial estimates × range of initial estimates: LRT_1,61_ = 4.88, p = 0.027). Also as in Experiment 1, the change in the group consensus estimate relative to the arithmetic mean of the initial estimates increased with the range of the initial estimates (neg. bin. GLMM: LRT_1,63_ = 14.32, p < 0.001). This trend was consistent across the different treatments (the treatment × range of initial estimates interaction was not significant: LRT_2,61_ = 0.57, p = 0.75).

As the groups progressed through the three tasks, the range of initial estimates changed differently depending on the proportion of black sweets (S9 Fig, question order × treatment: neg. bin. GLMM: LRT_4,60_ = 10.73, p = 0.030). There was little change in the range of initial estimates in the 48/200 treatment, a decline in ranges in the 94/190 treatment, and an increase in ranges in the 121/160 treatment. The errors in estimates for each individual declined as the trials progressed (S10 Fig, neg. bin. GLMM: LRT_2,417_ = 18.34, p < 0.001), suggesting that the process of repeated group interactions provided feedback that reduced the extent initial estimates were outliers. As each group carried out the task once for each treatment, we can also demonstrate that ranges of initial estimates were uncorrelated for groups between tasks (S11 Fig), so that disagreement of initial estimates was not a consistent trait of groups even within a short time scale and very similar tasks.

## Discussion

In the type of cognitive task used here, collective intelligence appears already well developed by the pre-teens and stable during adolescence, from at least 11 years of age. The changes in social and cognitive (specifically numerical) skills that occur during adolescence [7–9,12] do not appear to affect collective intelligence during this period of development. Especially when individuals disagreed regarding their (independently formed) initial estimates, the aggregation rule used by the groups to reach their group consensus estimate approximated the geometric mean. To our knowledge, this use of a geometric mean approximation in group consensus has not been demonstrated before, even in adults. Although this rule seems reasonable given the right-skewed overall distribution of initial estimates, aggregation rules such as the arithmetic mean, or even choosing a single one of the initial estimates, would be easier to implement. Studies of adults have in fact shown that individuals tend to choose a single estimate rather than taking the average when given multiple pieces of social information, despite averaging frequently being the more accurate strategy [25]. Our results show that groups of freely-interacting adolescents can implement a ‘wisdom of crowds’, where multiple, independent opinions are processed using a suitable averaging rule. However, further research is needed to determine which factors are needed for adolescents to approximate a geometric mean rule, such as face-to-face interaction [10], and how the pre-existing social friendship network of participants affect consensus decision making. Such an effect may explain differences between results in experiments 1 and 2, as groups were randomly formed across the school year in experiment 1, but participants were allowed to form their own groups in experiment 2. Additionally, although the average improvement in estimates after group discussion was not statistically different between females and males, when performance was measured as a binary ‘improved’ versus ‘not improved’, female participants were more likely to improve their estimate. This is consistent with previous work [26] where it was shown that the proportion of females in a group was positively related to performance in collective tasks, and that this could be explained by higher social sensitivity scores in females.

The lack of an effect of age, despite the greater constraints on cognitive and social skills, suggests that a heuristic that approximates the geometric mean provides a simple and robust method for aggregating individual estimates. Although other aggregation rules which we did not explore may equally match our results, human and non-human animals intuitively map numbers onto space in a way that appears to be logarithmic [27,28], providing a cognitive mechanism for how children at least as young as 11 years old can process social information in a way that approximates the geometric mean. The logarithmic representation of numbers provides a potential psychological process that downweighs but does not ignore outliers, with the result of the arithmetic mean of the logarithm of estimates being equivalent to the geometric mean of estimates (how estimates are integrated within groups has been recently reviewed elsewhere [29]). While the underlying mental representations that lead to this representation of numbers has been the primary focus of research [30], little attention has been paid to the advantages that such representations can bring. Our results suggest a socially-based benefit in a group decision making domain.

The effectiveness of a geometric rule of thumb in Experiment 1 is dependent on the right (positive) skewed distribution of initial estimates. Typically, where there was disagreement in initial estimates (high ranges), the distribution of estimates in the group was skewed (Fig 5b), thus even at the level of the group, the geometric mean is expected to be a successful strategy. In particular, it has been shown that log-normal distributions naturally arise and are common in tasks that involve high variance and where answers are limited to non-negative numbers [4,31], such as found in quantity estimation tasks, and we detect this nearly log-normal distribution in the results of Experiment 1. In tasks where there is aggregation of social and private information, the intuitive logarithmic-like processing of information is maintained. In fact, we have shown previously [23] that this integration can be modelled with a weighted geometric mean. The geometric mean might then be the optimal average, and is shown to be more accurate in these kinds of tasks [4]. This leads to two consequences: Firstly, groups naturally average their estimates approximating a geometric mean, because the logarithm of the geometric mean is the arithmetic mean of the logarithms. Secondly, additive noise in the logarithms will lead to Gaussian noise in the arithmetic mean of the logarithm of estimates, which translates to log-normal noise in the geometric mean of estimates.

As in adults [4], estimates made by individuals within groups converged after group discussion (in Experiment 1), and we also find in Experiment 2 that variability in initial estimates can decrease over repeated tasks although this only occurred in one of the three treatments (S9 Fig). The reduction in variability over repeated rounds of information sharing within each task demonstrated by Lorenz et al. [4] may thus generalise across consecutive tasks. Sharing of opinions during group discussion may allow individuals to learn that their initial estimate was too high or low relative to the rest of the group, which they attempt to correct in their initial estimate for the next task, reducing the overall range of the group’s initial estimates. For this feedback to be effective, it would depend on the tasks being fairly similar, as in our Experiment 2, although further work on this question is needed.

What also remains to be explored is how the improvement in decision making occurs [6], in particular how a rule that approximates the geometric mean is implemented. At one extreme, group discussion may only be needed to share the initial estimates, with a single individual then aggregating these estimates and giving the group’s consensus estimate. At the other extreme, group discussion itself may be necessary to process the initial estimates, with no one individual playing a disproportionate role. Analysing the verbal exchanges during the group discussion phase would be one approach to help determine the mechanisms; relatively simple measures such as the total amount of verbal discussion and how it is distributed between group members [26] may be adequate to explore correlations between group discussion and improved performance. Alternatively, the degree of information exchange could be manipulated, for example by having participants communicate via computers [4], although careful pilot experiments would be necessary to ensure young participants are able to use the technology and engage fully with the experiment.

## Materials and methods

### Participants

Experiment 1 used 147 participants aged between 11 and 19 years (mean ± s.d.: 13.7 ± 2.34, S1 Fig) from two secondary schools (Torquay Girls’ Grammar School (n = 45) and Okehampton College (n = 102), with 96 females and 51 males). Experiment 2 used 72 participants aged 12 to 14 years from Gordano School (32 females, 40 males). These sample sizes were effectively opportunistic, being limited by the time the schools made the students available away from their usual curriculum, and this is one of the limitations of working with children in a school setting. All schools are located in the south-west of the United Kingdom. Participation was voluntary, using an opt-in procedure. Initial consent was obtained from a parent or guardian of the participants through a letter sent to their home, which parents signed and sent back or replied by SMS to teachers. Consent was gained from participants after they were briefed by an experimenter, through signing a briefing form directly prior to the experiments. After testing, participants were debriefed about the aims of the experiments and offered the opportunity to ask questions or to withdraw their data. In both experiments all data were kept completely anonymous and are available as S1 and S2 Datasets. All procedures were in accordance with British Psychological Society guidelines for ethics in research and were approved by the University of Exeter Psychology Research Ethics Committee (ref: 2010/106) and the University of Bristol Faculty of Science Research Ethics Committee (ref: 2609134005).

### Procedure, Experiment 1

49 groups of 3 school children were asked to guess the number of sweets in a jar (S2 Fig), with experiments taking place in 2011. Within their school year, participants were randomly assigned to same-gender groups of three. Participants were first asked to provide details of their school year, age and gender. The experimenter presented the clear, octagonal jar (diameter = 9.5 cm, height = 19 cm) of 57 toffee sweets (S2 Fig) to the group. Participants were asked to individually estimate the number of sweets in the jar and write their answer on the answer sheet, without conferring or otherwise exchanging information between themselves. Following this, participants were asked to discuss the number of sweets in the jar as a group, and decide on a group estimate which they felt was the most accurate. The time for discussion was limited to five minutes, although all groups made their consensus estimate more quickly than this. Participants were then asked to make a final individual estimate, which could again vary between group members. The jar remained visible throughout. Participants were allowed to pick up the jar, but the lid was sealed so the contents could not be removed. The same jar of sweets was used for all groups. Separate answer sheets were used for each stage and, after each estimate, participants handed these answer slips back to experimenters. After each stage, participants were asked to rate their confidence that their answer was correct on a written Likert confidence scale. The scale ranged between 1 and 7: 1 represented “not at all confident” while 7 represented “very confident”. However, as these self-rated confidences were not found in our preliminary analyses to relate to accuracy or how much individuals changed their estimates between stages (S12 Fig), they were not analysed further.

### Procedure, Experiment 2

We presented participants aged 12 to 14 years old jars containing a mix of black sweets and white sweets (S2 Fig) and they were initially asked to estimate the number of black sweets without communicating with their group, and then to give a group consensus estimate after group discussion. The total number of sweets per jar was written on top of each jar, providing an upper limit to the total possible number of black sweets (unlike the task in Experiment 1), and hence limiting the extent of outliers within groups. Jars contained one of three proportions of black sweets (48/200, 94/190, 121/160), and 24 groups of three participants were tested once in each of these tasks, which also allowed investigation of testing order effects, such as how estimates changed over repeated trials.

Experiments took place in 2014. Participants were asked to form groups of 3 (mixed gender groups were allowed), and presented with a single jar (diameter = 7.5 cm, height = 11 cm) containing a mixture of black sweets and white sweets. The proportion of black sweets varied between treatments (48/200, 94/190, 121/160), and participants were told the total number of sweets in their jar, which was also written on the lid of the jar. Although the total number of sweets changed across treatments, the approximate volume of the sweets (and size of the jars) was constant as the white sweets were slightly smaller than the black sweets. Participants were asked to estimate how many black sweets were in the jar without working with the rest of the group. They were then asked to give a group consensus estimate after a period of group discussion. Participants were allowed to pick up the jar, but the lids were sealed so the contents could not be removed. Once the first set of estimates were given, a jar with a different proportion was presented and the task repeated. In total, each group made estimates for jars with all three proportions of black sweets in a randomised order, except one group which did not complete the 121/160 treatment. Separate answer sheets were used for each treatment. Participants were then asked to give their gender, age and school year before all answer sheets were collected.

### Statistical analysis: Linear Models Experiment 1

These tests were conducted in R v3.2.3. Due to the right-skewed nature of the response variable data, negative binomial error distributions were used throughout, with one exception in Experiment 1 as detailed below where the response was a binary variable, in General Linear Mixed Models (using the glmmadmb function in the glmmADMB package) and General Linear Models (using the glm function in the stats package). The significance of each term was tested using the drop1 function in the stats package, which uses Likelihood Ratio Tests. In models with interaction terms, the interaction(s) with a p value > 0.05 were removed and models were rerun to test whether any remaining interaction terms and main effects had a significant effect on the response variable. The random effects specified are random intercepts. Models and their results are summarised in S1 Table (Experiment 1) and S3 Table (Experiment 2).

The absolute error of each participants’ estimates (i.e. the absolute value of the estimate minus the correct number of sweets) was analysed as a function of their age, gender, and the stage at which they made the estimate (before group discussion, the group discussion estimate or after group discussion), as well as all two-way interactions between these variables. Participant identity nested in group identity was included as a random effect. A binomially distributed GLMM was used to analyse the probability that a participant improved their estimate after group discussion (i.e. 0 for an estimate that did not improve, 1 for an estimate that did improve) as a function of the stage (whether the estimate was given as the group consensus estimate or the post-discussion individual estimate), and the age and gender of the participants. Again, all two-way interactions between these three variables were initially included with participant identity nested in group identity included as a random effect. The absolute error in the groups’ consensus estimates was analysed as a function of mean age of participants in the group, the absolute error in the (arithmetic) mean of their initial estimates and the range of these estimates within the group. Two-way interactions between these variables were included, as was the main effect of gender, and there were no random terms. The % change in the group consensus estimate versus the mean of the initial estimates is calculated as the absolute value of Group estimate/Mean initial estimate×100–100. This was analysed as a function of mean age, gender and range of initial estimates as main effects, and there were no random terms. The effect of the range of initial estimates on the observed consensus group estimate, arithmetic and geometric means and mean of the two closer initial estimates was analysed with the value of the estimate as the response variable and the range, estimate type (e.g. geometric mean) and mean age in the group as explanatory variables, including their two-way interactions, and gender as a main effect. Group identity was included as a random effect. The post-discussion estimate was analysed as a function of individual participants’ age, gender and initial estimate as main effects; this was repeated using the log10 initial estimate in a separate model. Group identity was included as a random effect. Finally, the absolute error of different aggregation rules, as well as the error in observed group consensus estimate, was analysed as a function of the aggregation rule, mean age, and gender as main effects with group identity as a random effect.

### Statistical analysis: Linear Models Experiment 2

These tests were also conducted in R v3.2.3 using the same statistical model functions as in Experiment 1. The absolute error of each participant’s estimates was analysed as a function of treatment (the proportion of black sweets in the jar), whether the estimate was before group discussion or the group consensus estimate, and the interaction between these two variables. The order of the treatment (1st, 2nd or 3rd) was included as a main effect, and participant identity nested in group identity was included as a random effect. The absolute error in the group estimate was analysed as a function of the absolute error in the mean of the pre-discussion individual estimates, the range of these estimates, and the two-way interaction between these terms. Treatment and treatment order were included as main effects, and group identity as a random effect. As in Experiment 1, the % change in the group consensus estimate versus the arithmetic mean of the initial estimates was calculated as the absolute value of Group estimate/Mean initial estimate×100–100. This was analysed as a function of treatment, the range of the initial estimates and the interaction term between these effects. Treatment order was included as a main effect and group identity as a random effect. The range of the initial estimates in each group was analysed as a function of treatment, treatment order and the interaction between these terms, with group identity as a random effect.

## Supporting information

S1 Methods Log-likelihood of simple aggregation rules, the noisy geometric mean model, and confidence intervals for frequencies of the aggregation rules using the noisy geometric mean model. (PDF)

S1 Fig The age distribution of participants in Experiment 1, correlations within groups, and distribution of individual initial estimates. (PDF)

S2 Fig The jars of sweets used in Experiment 1 and Experiment 2. (PDF)

S3 Fig The relationship between the range of initial estimates and estimates given by the group or calculated from initial estimates in Experiment 1. (PDF)

S4 Fig Fits of different aggregation rules to the observed data at various levels of added noise in Experiment 1. (PDF)

S5 Fig The relationship between individual estimates before and after group discussion in Experiment 1. (PDF)

S6 Fig The use and consequence of different aggregation rules for different thresholds that define groups as having a low or high range in Experiment 1. (PDF)

S7 Fig Distribution of individual initial estimates in Experiment 2. (PDF)

S8 Fig The effect of disagreement (range) in initial estimates on improving group estimates in Experiment 2. (PDF)

S9 Fig The effect of question order and treatment on the disagreement (range) of initial estimates in Experiment 2. (PDF)

S10 Fig The effect of question order on the absolute error of (initial and group consensus) estimates in Experiment 2. (PDF)

S11 Fig Correlations across treatments in the range of individual initial estimates per group. (PDF)

S12 Fig The relationship between participants’ self-rated confidence and error and the change in individuals estimates between stages. (PDF)

S13 Fig The distribution of the difference between the group estimate and the mean of initial estimates. (PDF)

S1 Table Summary of statistical tests in Experiment 1. (PDF)

S2 Table Absolute error of the geometric rule compared to the other aggregation rules and the observed consensus estimates (Experiment 1), for all groups, and then with the groups split by those with low (≤40) and high ranges (>40). (PDF)

S3 Table Summary of statistical tests from Experiment 2. (PDF)

S1 Dataset Data from Experiment 1 (.csv format). (CSV)

S2 Dataset Data from Experiment 2 (.csv format). (CSV)
