# Supplementary material for: Adolescents show collective intelligence which can be driven by a geometric mean rule of thumb
Source: PLoS One. 2018 Sep 24;13(9):e0204462. doi: 10.1371/journal.pone.0204462 (PMC6152954; doi:10.1371/journal.pone.0204462)
Supplement: S1 Methods — (PDF) [file pone.0204462.s001.pdf]

## S1 Methods

### Log-likelihood of simple aggregation rules

MATLAB R2014a was used for the analyses in S1 Methods. We computed the log-likelihood of each of the proposed aggregation rules as the logarithm of the likelihood $L$ that the group consensus estimates are generated by a noisy computation of the rule. This was modeled by considering probability distributions centered at the values of the rule computed with the individual initial estimates of each group:

$$L\left(R \mid \{c_i\}_{i=1}^{n}\right) = f\left(\{c_i\}_{i=1}^{n} \mid R\right) = \prod_{i=1}^{n} f_i\left(c_i \mid r_i\right) \tag{1}$$

where $R$ denotes one particular rule, $\{c_i\}_{i=1}^{n}$ is the set of observed discussion estimates agreed by each of the $n$ groups, $r_i$ is the exact value that the rule takes for the $i$-th group, and $f_i$ was assumed to be a normal or log-normal with parameters $r_i$ and $\sigma$. For each of the considered rules, we covered a wide range of possible values for $\sigma$ to test for the dependence of the likelihood on the level of noise considered. The geometric mean was found to be the most likely rule to be generating the experimentally observed group estimates (Fig 4), and the log-normal noise provided a higher log-likelihood value than the Gaussian noise.

### The noisy geometric mean model

We modelled groups of three subjects reaching a consensus from their initial individual estimates. Specifically, we considered that, given three estimates $x_1$, $x_2$ and $x_3$, the group gave a consensus estimate $c$ sampling from some probability density function:

$$f\left(c \mid x_1, x_2, x_3\right). \tag{2}$$

In the section 'Log-likelihood of simple aggregation rules' above, we show that of all the rules proposed, the one with a higher likelihood of producing the experimental results is the geometric mean (Fig 4):

$$f\left(c \mid g\right), \tag{3}$$

with

$$g \equiv \left(x_1 x_2 x_3\right)^{1/3} \tag{4}$$

the geometric mean of the three initial estimates. The noise that produces the consensus value to deviate from the geometric mean can have a Gaussian form,

$$f\left(c \mid g\right) = \frac{1}{\sigma\sqrt{2\pi}} e^{-\frac{1}{2}\left(\frac{c-g}{\sigma}\right)^2}, \tag{5}$$

or a log-normal form,

$$f\left(c \mid g\right) = \frac{1}{c\sigma\sqrt{2\pi}} e^{-\frac{1}{2}\left(\frac{\log(c)-\log(g)}{\sigma}\right)^2}. \tag{6}$$

We favoured the log-normal option for two reasons. First, it provides a higher log-likelihood value. Second, it is in greater agreement with the logarithmic-like effect shown in the main text and the nearly log-normal shape of the distribution (there is a statistically significant deviation from normality: Kolmogorov-Smirnov test over the z-scored logarithm of individual initial estimates, $p = 0.035$; S1D Fig). We provide further justification for the log-normal noise in S13 Fig.

The $\sigma$ parameter of the noise function can be established in at least two different ways. One is to use the value that provides a higher log-likelihood (Eq. (1)). The other is to compute the standard deviation of the set of 49 experimental values $\{\Delta_i\}_{i=1}^{49}$, where $\Delta_i$ is defined as the difference in logarithms from the geometric mean $g_i$ of the $i$-th group's initial estimates to their group consensus estimate:

$$\Delta_i \equiv \log(c_i) - \log(g_i) \quad (7)$$
.

Both methods provide similar results (discrepancies of less than 1%).

The set of simulated consensus estimates $\{c_i\}_{i=1}^{49}$ will be thus a 49 dimension random variable, produced by the set of 49 probability density functions $\{f_i\}_{i=1}^{49}$, with

$$f_i(c_i \mid g_i) = \frac{1}{c_i \sigma \sqrt{2\pi}} e^{-\frac{1}{2}\left(\frac{\log(c_i) - \log(g_i)}{\sigma}\right)^2} \quad (8)$$
,

for the case of log-normal noise. Note that uncorrelation between groups is assumed.

A third way of setting the noise would be to consider for each group a different standard deviation, estimated via the $\Delta_i$ value defined in Eq. (7):

$$f_i(c_i \mid g_i) = \frac{1}{c_i \Delta_i \sqrt{2\pi}} e^{-\frac{1}{2}\left(\frac{\log(c_i) - \log(g_i)}{\Delta_i}\right)^2} \quad (9)$$
,

but the previous two methods are in more agreement with the idea of a single 'noisy' rule generating the experimental results.

**Confidence intervals for frequencies of the aggregation rules using the noisy geometric mean model**

We estimated the $\sigma$ parameter to be used in Eq. (8) computing the standard deviation of the set $\{\Delta_i\}_{i=1}^{49}$ of values obtained applying Eq. (7) to the 49 experimental groups. With this standard deviation parameter, we generated a set $\{\xi_i\}_{i=1}^{49}$ of noise values to be added to each of the 49 geometric mean values $g_i$ obtained from the initial estimates of the groups (Eq. (4)):

$$\log(c_i) = \log(g_i) + \xi_i \quad (10)$$
,

with each $\xi_i$ value sampled from a Gaussian $N(0,\sigma)$. This way, we obtained a set $\{c_i\}_{i=1}^{49}$ of 49 simulated consensus values, and then determined for each group which rule (Fig 4) was closest to their simulated group consensus estimate. For the $i$-th group, the set $\{r_{ij}\}_{j=1}^{8}$ of values that each of the 8 considered rules take was computed from the pre-consensus values obtained in the real experiment. To assign one or more rules from the set $\{r_{ij}\}_{j=1}^{8}$, a minimum distance criterion was applied. For that, the set $\{d_{ij}\}_{j=1}^{8}$ of distances from the generated consensus value to that of the rule,

$$d_{ij} \equiv \left| c_i - r_{ij} \right| , \qquad (11)$$

was computed. Then, the $j$-th rule was classified as followed by the $i$-th group if

$$d_{ij} = \min \{d_{ij}\}_{j=1}^{8} . \qquad (12)$$

The frequency $q_j$ of the $j$-th rule was not computed simply as the number of groups for which Eq. (12) was fulfilled by $d_{ij}$, because for some groups Eq. (12) was fulfilled by not one but $n_i$ rules. Instead, the contribution of the $i$-th group to each of the rules was determined as

$$q_{ij} = \begin{cases} 1/n_i & \text{if } d_{ij} \text{ fulfills Eq. S12} \\ 0 & \text{otherwise} \end{cases} , \qquad (13)$$

and then the frequencies were actually computed as

$$q_j = \sum_{i=1}^{49} q_{ij} . \qquad (14)$$

Note that, for each group, we did not sort the rules in ascending numerical order, but keeping always the same operation performed with the three pre-consensus values in the

same position across groups. To turn the frequencies $q_i$ into probabilities $p_i$, we divided each by the total number of rules considered:

$$p_i = \frac{q_i}{8} . \tag{15}$$

We repeated 10,000 times the process we have detailed, obtaining for each rule a sample distribution of 10,000 frequencies (and probabilities computed with Eq. (15) when required) compatible with the noisy geometric model. For each of these distributions, the mean and 2.5 and 97.5 percentiles were computed. This way, we obtained for each rule the mean of compatible probabilities (blue line in Fig 5a,c), and the limits that contain 95% of compatible probabilities (upper and lower limits of the shaded areas in Fig 5a,c).
