# Supplementary material for: Adolescents show collective intelligence which can be driven by a geometric mean rule of thumb
Source: PLoS One. 2018 Sep 24;13(9):e0204462. doi: 10.1371/journal.pone.0204462 (PMC6152954; doi:10.1371/journal.pone.0204462)
Supplement: S1 Fig — (PDF) [file pone.0204462.s002.pdf]

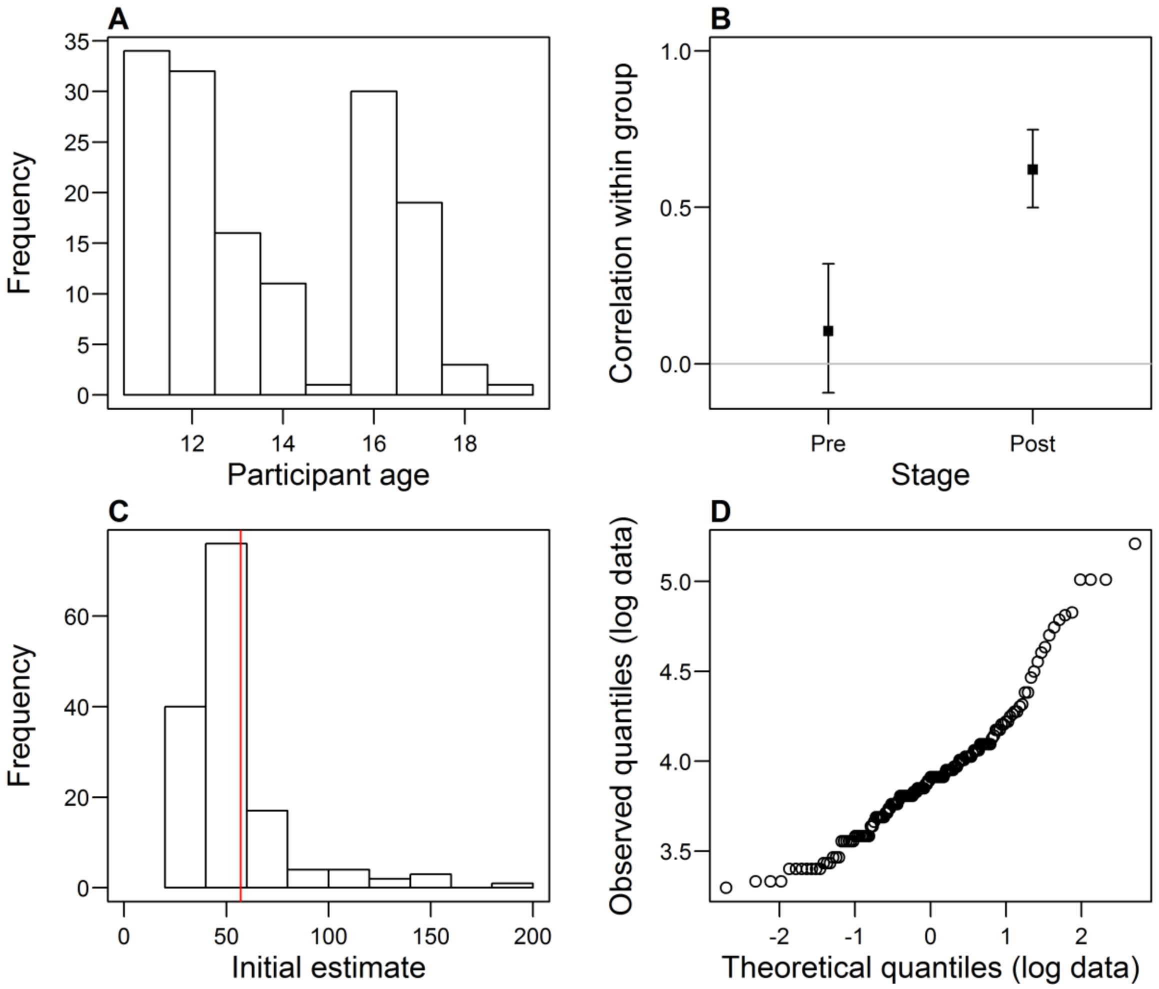

S1 Fig. The age distribution of participants in Experiment 1, correlations within groups, and distribution of individual initial estimates. (**A**) The number of participants at each age in the sample of Experiment 1. (**B**) To determine whether individual initial estimates were independent within groups and post-discussion estimates correlated within groups, we carried out a randomisation test. In each of 10,000 iterations, we randomly selected an individual from each group as individual 1 and another (without replacement) as individual 2. The correlation between the estimates of individual 1 and 2 was then determined for pre and post discussion estimates separately. The means (solid squares) and 95% confidence intervals (error bars) for the two distributions of correlation coefficients are shown. The overlap of the 95% confidence intervals for initial estimates with a correlation of zero suggests estimates were independently made at this stage. In contrast, estimates were positively correlated

within groups after discussion. (**C**) The frequency distribution of individual initial estimates. The correct value, 57 sweets, is shown by the red vertical line. (**D**) A quantile-quantile plot of the log10 individual initial estimates; this demonstrates that the distribution of initial estimates were not log normal (as the points do not fall on a straight line).
