# Supplementary material for: Adolescents show collective intelligence which can be driven by a geometric mean rule of thumb
Source: PLoS One. 2018 Sep 24;13(9):e0204462. doi: 10.1371/journal.pone.0204462 (PMC6152954; doi:10.1371/journal.pone.0204462)
Supplement: S2 Fig — (PDF) [file pone.0204462.s003.pdf]

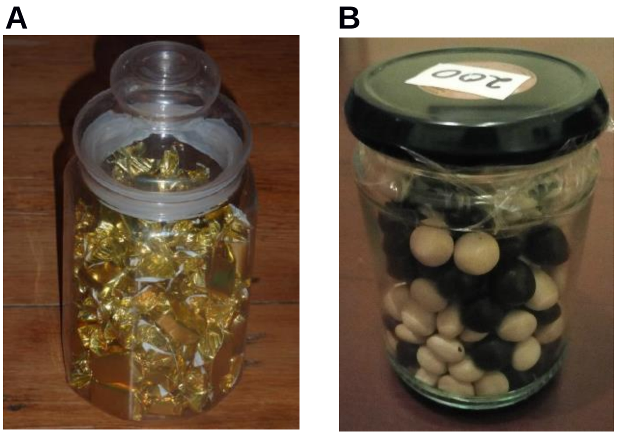

S2 Fig. The jars of sweets used in Experiment 1 and Experiment 2. (**A**) The jar contained 57 golden foil-wrapped toffees. (**B**) The jar containing a mixture of black and white sweets, in the proportion 48/200.
