# Supplementary material for: Adolescents show collective intelligence which can be driven by a geometric mean rule of thumb
Source: PLoS One. 2018 Sep 24;13(9):e0204462. doi: 10.1371/journal.pone.0204462 (PMC6152954; doi:10.1371/journal.pone.0204462)
Supplement: S3 Fig — (PDF) [file pone.0204462.s004.pdf]

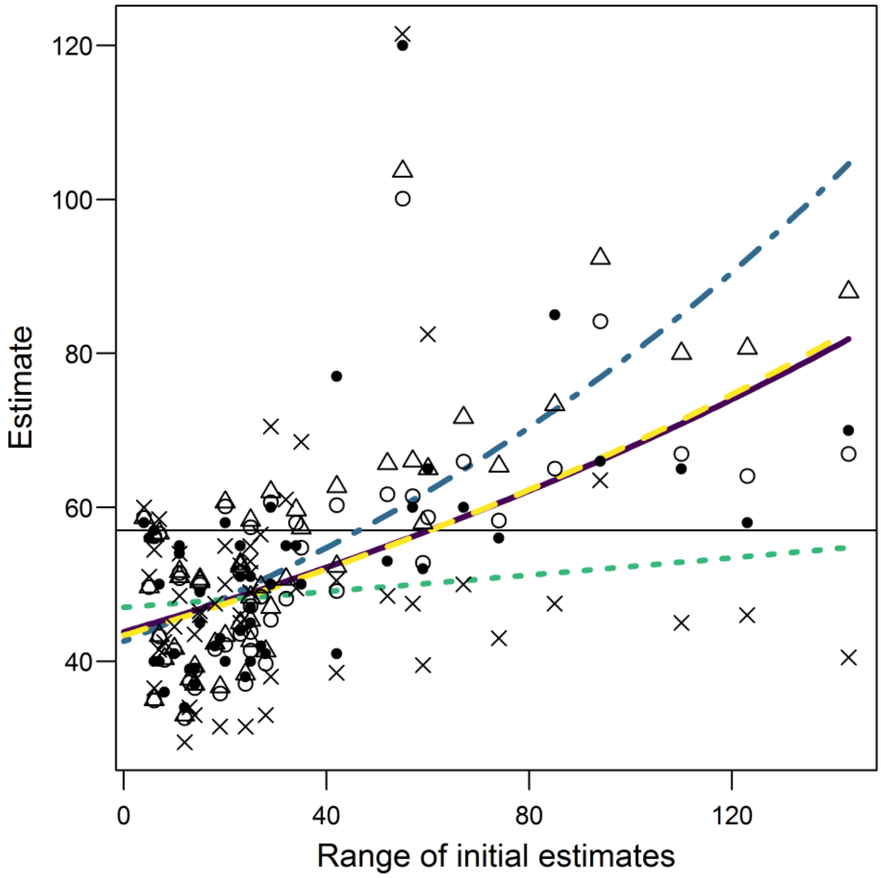

S3 Fig. The relationship between the range of initial estimates and estimates given by the group or calculated from initial estimates in Experiment 1. Shown is the group consensus estimate (dark purple solid line and filled circles), the arithmetic mean of all initial estimates (dark blue dashed-dotted line and triangles), the arithmetic mean of the two initial estimates that were closest to one another (green dotted line and crosses) and the geometric mean of all initial estimates (yellow dashed line and open circles). The solid horizontal line indicates the number of sweets in the jar, 57. The fitted lines are calculated from the neg. bin. GLMM coefficients, including the significant interaction and main effects of gender and mean age that are fixed at their mean values in the data set. Overall there is a significant interaction between the type of estimate and the range of initial estimates, i.e. their slopes differ significantly (neg. bin. GLMM: $LRT_{3,184} = 34.016$, $p < 0.001$). The positive relationship of the range on the arithmetic mean and minimal effect on the mean of the two closer estimates suggests that large ranges were due to a single value greater than the two

closer estimates (an outlier), which is consistent with the overall distribution of initial estimates (S1C,D Fig). The groups' consensus estimates had an intermediate trend, with a significantly steeper relationship with the range than the mean of the two estimates that were closest to one another, but a relationship less steep than expected from the mean of all initial estimates. This shows that the weight given to outlier estimates was reduced compared to simple averaging of all initial estimates but not dismissed entirely in the group consensus decision. The overall relationship with the group range for groups' consensus estimates were close to that of the geometric mean of the initial estimates.
