# Supplementary material for: Adolescents show collective intelligence which can be driven by a geometric mean rule of thumb
Source: PLoS One. 2018 Sep 24;13(9):e0204462. doi: 10.1371/journal.pone.0204462 (PMC6152954; doi:10.1371/journal.pone.0204462)
Supplement: S4 Fig — (PDF) [file pone.0204462.s005.pdf]

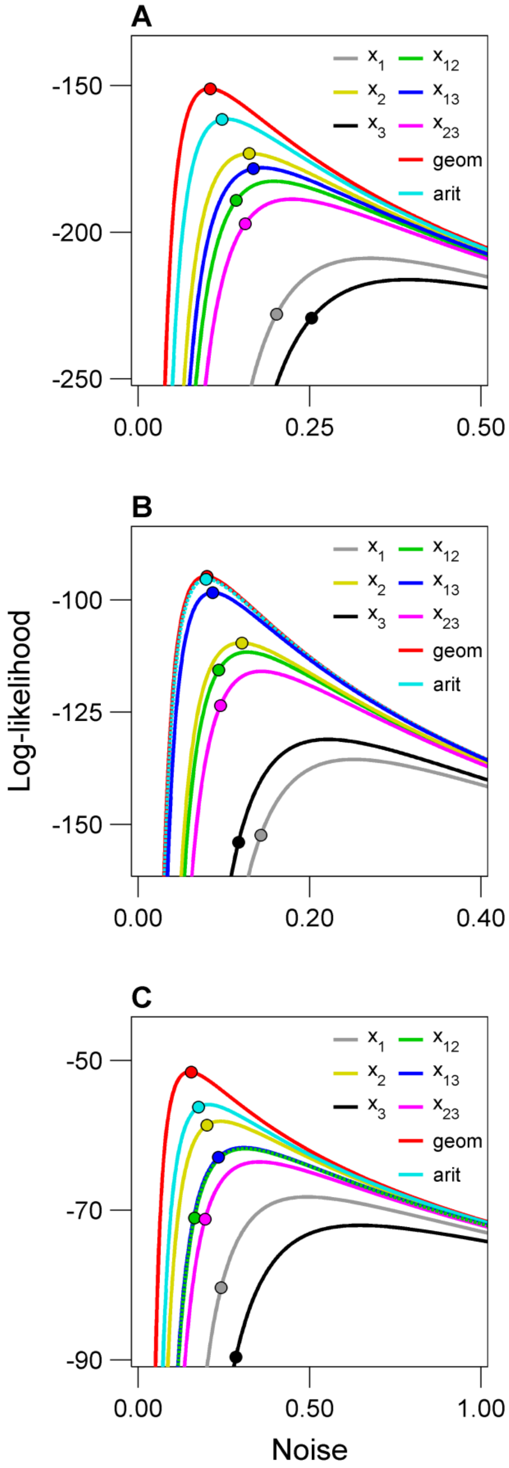

S4 Fig. Fits of different aggregation rules to the observed data at various levels of added noise in Experiment 1. (**A**) Log-likelihood of the observed consensus values being produced from the exact value of each rule tested for a range of noise values (curves). Highlighted are the log-likelihood values corresponding to the observed level of deviation around the rule (circles). (**B**) As **A**, but only for groups with a low range (≤40) of estimates. (**C**) As **A**, but

only for groups with a high range (>40) of estimates. The rules tested are: the lowest estimate (x_1), mean of the lowest and median estimate (x_{12}), the median (x_2), geometric (geom) and arithmetic (arit) means, mean of the lowest and highest estimates in each group (x_{13}), mean of median and highest estimate (x_{23}), and highest estimate (x_3). At each level of noise, the geometric mean consistently provides amongst the best fits to the observed data.
