# Supplementary material for: Adolescents show collective intelligence which can be driven by a geometric mean rule of thumb
Source: PLoS One. 2018 Sep 24;13(9):e0204462. doi: 10.1371/journal.pone.0204462 (PMC6152954; doi:10.1371/journal.pone.0204462)
Supplement: S5 Fig — (PDF) [file pone.0204462.s006.pdf]

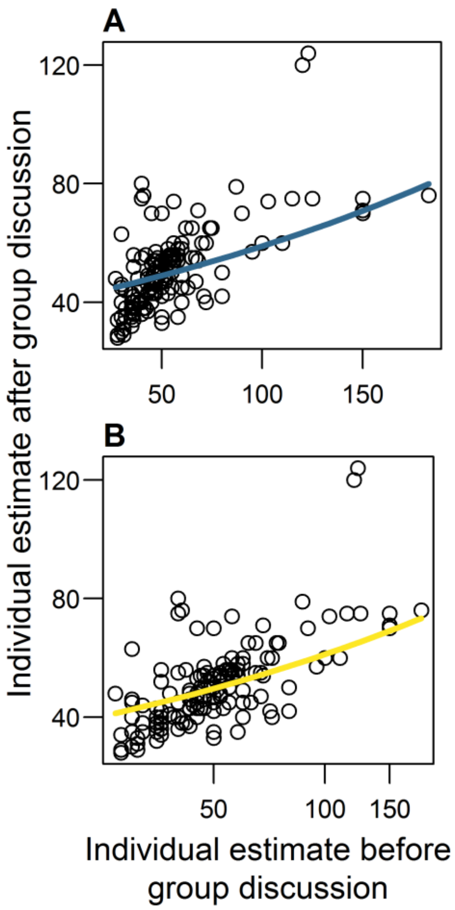

S5 Fig. The relationship between individual estimates before and after group discussion in Experiment 1. (**A**) shows the post-discussion estimate plotted against the untransformed initial estimate, while (**B**) plots it against the log10 initial estimate. The lines of best fit are from neg. bin. GLMMs with either the untransformed (**A**, dark blue line) or log10 transformed (**B**, yellow line) initial estimate as the explanatory variable. The main effects of gender and age are fixed at their mean values in the data set.
