# Supplementary material for: Adolescents show collective intelligence which can be driven by a geometric mean rule of thumb
Source: PLoS One. 2018 Sep 24;13(9):e0204462. doi: 10.1371/journal.pone.0204462 (PMC6152954; doi:10.1371/journal.pone.0204462)
Supplement: S6 Fig — (PDF) [file pone.0204462.s007.pdf]

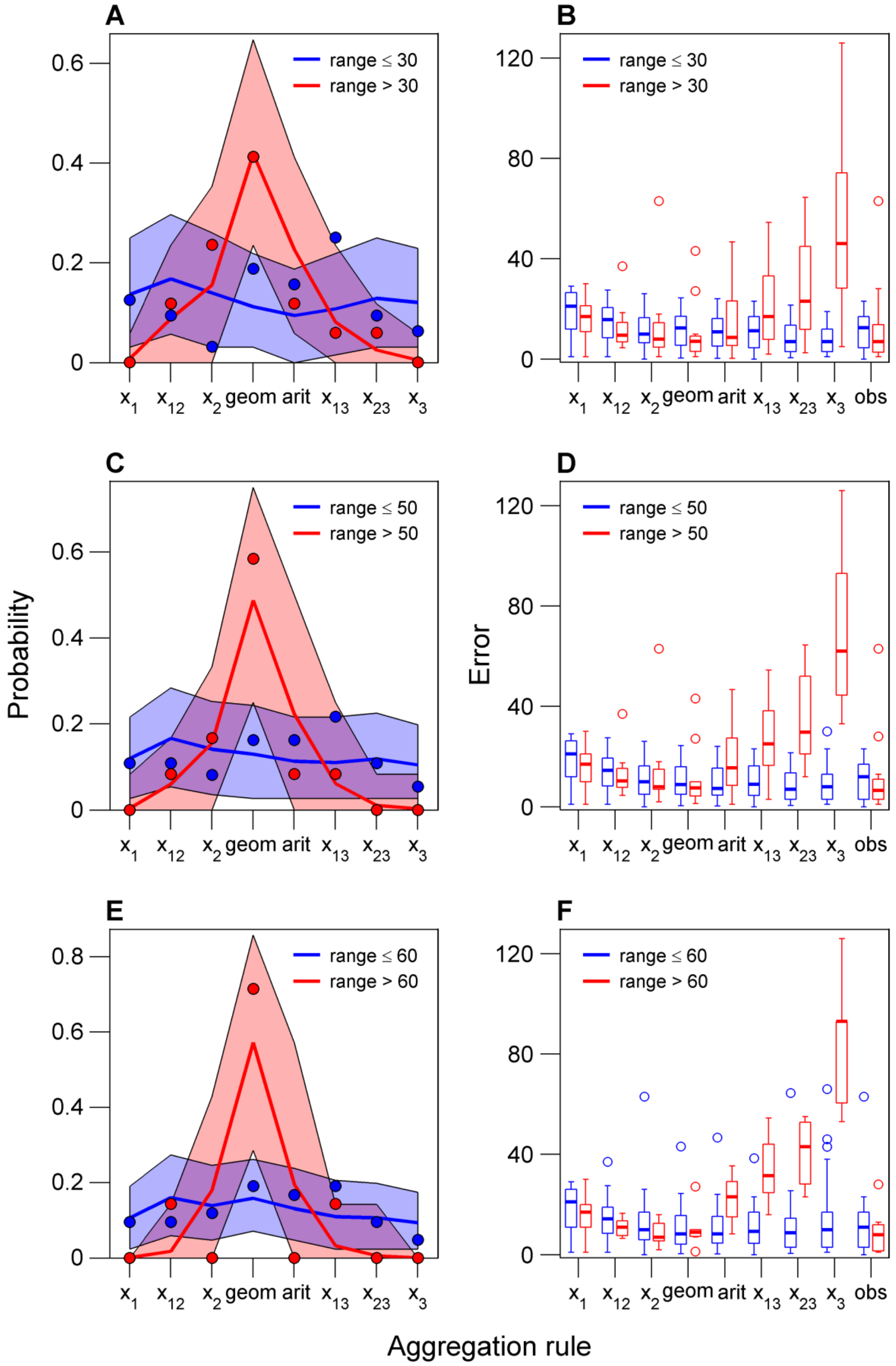

S6 Fig. The use and consequence of different aggregation rules for different thresholds that define groups as having a low or high range in Experiment 1. The thresholds tested are 30 (**A**,**B**), 50 (**C**,**D**) and 60 (**E**,**F**). Plotting as in Fig 5.
