# Supplementary material for: Adolescents show collective intelligence which can be driven by a geometric mean rule of thumb
Source: PLoS One. 2018 Sep 24;13(9):e0204462. doi: 10.1371/journal.pone.0204462 (PMC6152954; doi:10.1371/journal.pone.0204462)
Supplement: S7 Fig — (PDF) [file pone.0204462.s008.pdf]

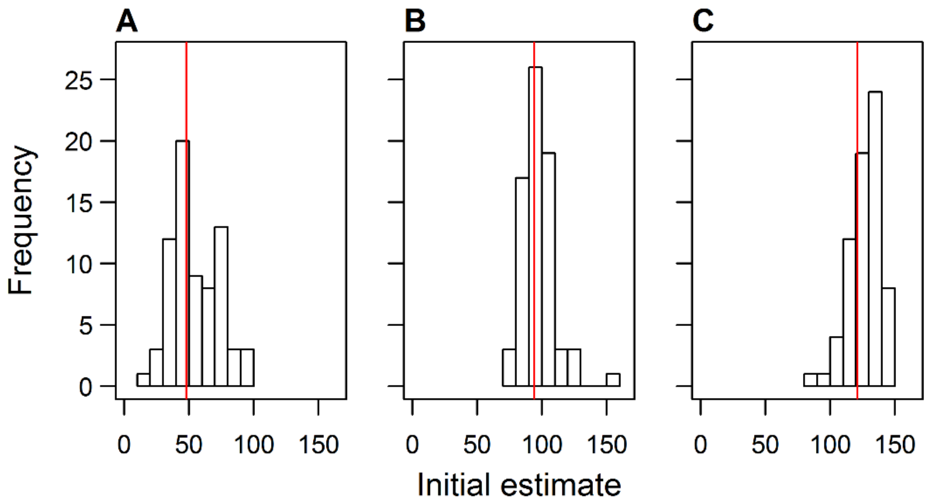

S7 Fig. Distribution of individual initial estimates in Experiment 2. The distributions of estimates (how many black sweets were in each jar) depended on the proportion of black sweets (**A**: 48/200, **B**: 94/190, **C**: 121/160). The correct value for each treatment is shown by the red vertical lines.
