# Supplementary material for: Adolescents show collective intelligence which can be driven by a geometric mean rule of thumb
Source: PLoS One. 2018 Sep 24;13(9):e0204462. doi: 10.1371/journal.pone.0204462 (PMC6152954; doi:10.1371/journal.pone.0204462)
Supplement: S8 Fig — (PDF) [file pone.0204462.s009.pdf]

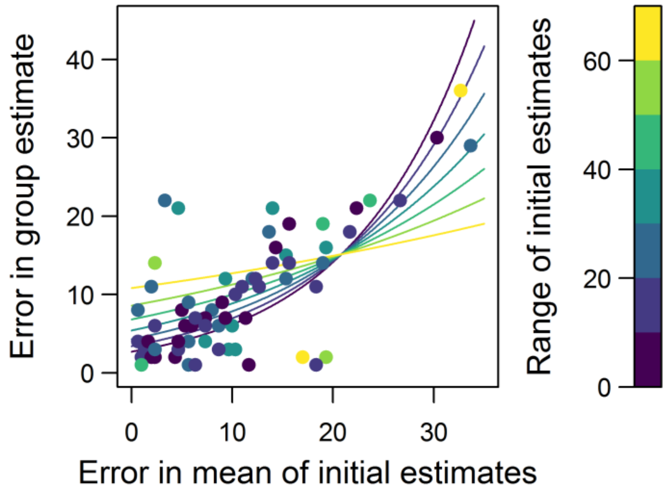

S8 Fig. The effect of disagreement (range) in initial estimates on improving group estimates in Experiment 2. As in Fig 2 (Experiment 1), disagreement is measured as the range of initial estimates in each group in each treatment. The colours represent this range, binned every ten units. Coloured lines are fits for each range interval, calculated from the GLMM coefficients. The main effect of treatment order is included in the fitted values, fixed at the mean value in the data. In groups with larger ranges, the positive relationship between errors in initial and group estimates reduces.
