# Supplementary material for: Adolescents show collective intelligence which can be driven by a geometric mean rule of thumb
Source: PLoS One. 2018 Sep 24;13(9):e0204462. doi: 10.1371/journal.pone.0204462 (PMC6152954; doi:10.1371/journal.pone.0204462)
Supplement: S9 Fig — (PDF) [file pone.0204462.s010.pdf]

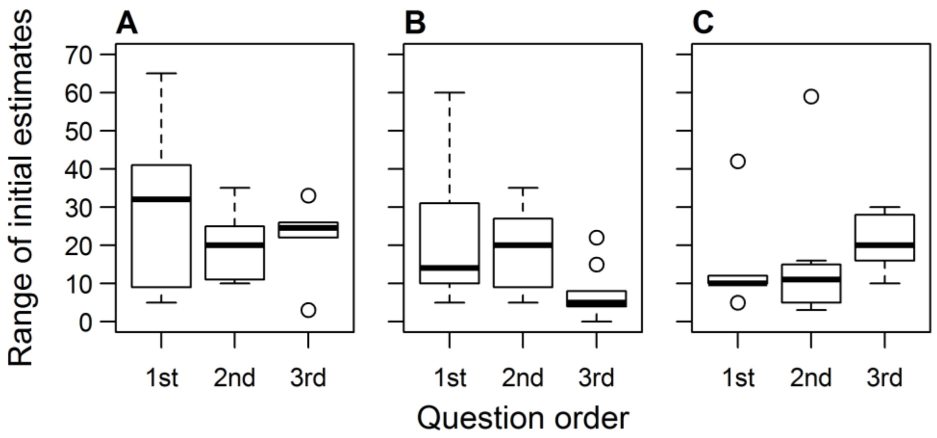

S9 Fig. The effect of question order and treatment on the disagreement (range) of initial estimates in Experiment 2. Data are panelled by treatment (proportion of black sweets): 48/200 (A), 94/190 (B) and 121/160 (C). The box plots show the median (thick black lines), interquartile range (enclosed by the boxes), 1.5 × the interquartile range beyond the boxes (whiskers) and outliers beyond the whiskers (open circles).
