# Supplementary material for: Adolescents show collective intelligence which can be driven by a geometric mean rule of thumb
Source: PLoS One. 2018 Sep 24;13(9):e0204462. doi: 10.1371/journal.pone.0204462 (PMC6152954; doi:10.1371/journal.pone.0204462)
Supplement: S10 Fig — (PDF) [file pone.0204462.s011.pdf]

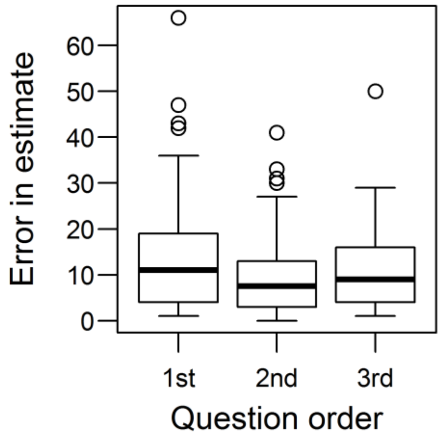

S10 Fig. The effect of question order on the absolute error of (initial and group consensus) estimates in Experiment 2. The box plots show the median (thick black lines), interquartile range (enclosed by the boxes), 1.5 × the interquartile range beyond the boxes (whiskers) and outliers beyond the whiskers (open circles).
