# Supplementary material for: Adolescents show collective intelligence which can be driven by a geometric mean rule of thumb
Source: PLoS One. 2018 Sep 24;13(9):e0204462. doi: 10.1371/journal.pone.0204462 (PMC6152954; doi:10.1371/journal.pone.0204462)
Supplement: S11 Fig — (PDF) [file pone.0204462.s012.pdf]

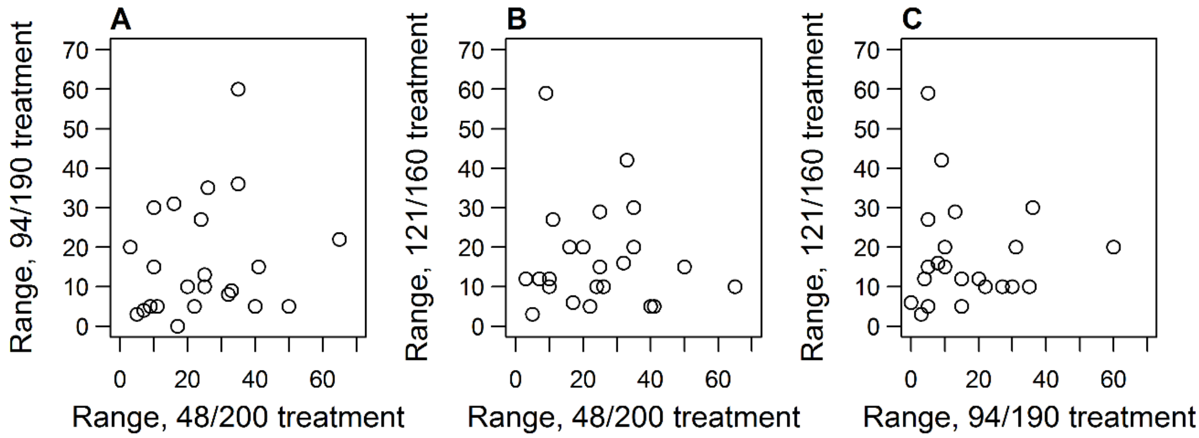

S11 Fig. Correlations across treatments in the range of individual initial estimates per group. The ranges for each group from the 48/200 treatment is plotted against the ranges for those groups in the 94/190 treatment (A) and 121/160 treatment (B), and ranges from the 94/190 treatment are plotted against those from the 121/160 treatment (C). In all cases, there is no significant correlation in group ranges across treatments (Spearman's rank correlation: A: $r_s = 0.27$, $p = 0.21$; B: $r_s = 0.0090$, $p = 0.97$; C: $r_s = 0.20$, $p = 0.37$), suggesting that individuals in some groups do not consistently agree with one another while in other groups individuals disagree.
