# Supplementary material for: Adolescents show collective intelligence which can be driven by a geometric mean rule of thumb
Source: PLoS One. 2018 Sep 24;13(9):e0204462. doi: 10.1371/journal.pone.0204462 (PMC6152954; doi:10.1371/journal.pone.0204462)
Supplement: S12 Fig — (PDF) [file pone.0204462.s013.pdf]

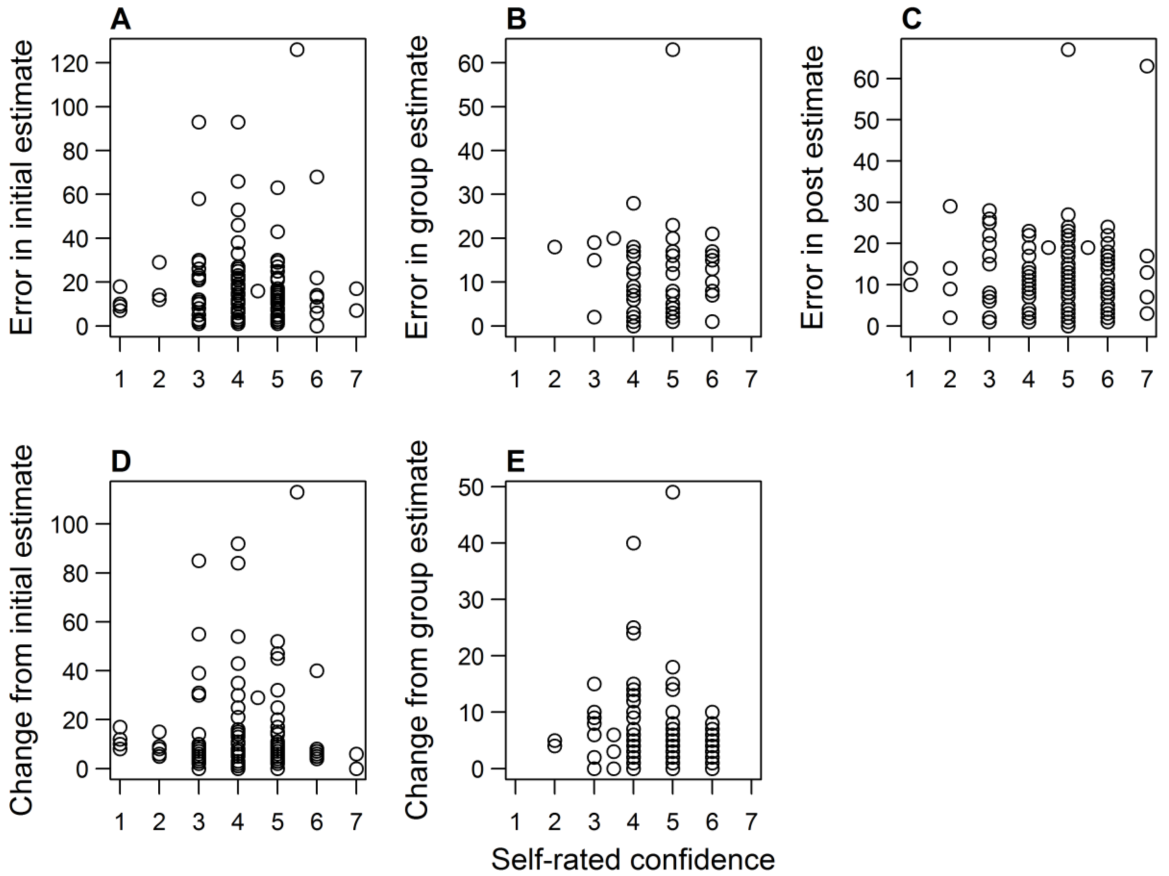

S12 Fig. The relationship between participants' self-rated confidence and error and the change in individuals estimates between stages. Shown is the relationship between the confidence rating and absolute error in the estimate at the corresponding stage (e.g. error in the initial estimate plotted against the confidence rating given for that initial estimate, **A**-**C**) and between the confidence rating and how much the estimate changed between the corresponding estimate and the estimate in the next stage (**D**: from the initial to the group estimate and **E**: from the group to the post-discussion estimate). Confidence ratings in their estimate of the number of sweets were given by individuals (initial and post estimate confidence) or as a single group (group estimate confidence) on a written Likert confidence scale. The scale ranged between 1 and 7: 1 represented "not at all confident" while 7 represented "very confident".
