# Supplementary material for: Adolescents show collective intelligence which can be driven by a geometric mean rule of thumb
Source: PLoS One. 2018 Sep 24;13(9):e0204462. doi: 10.1371/journal.pone.0204462 (PMC6152954; doi:10.1371/journal.pone.0204462)
Supplement: S13 Fig — (PDF) [file pone.0204462.s014.pdf]

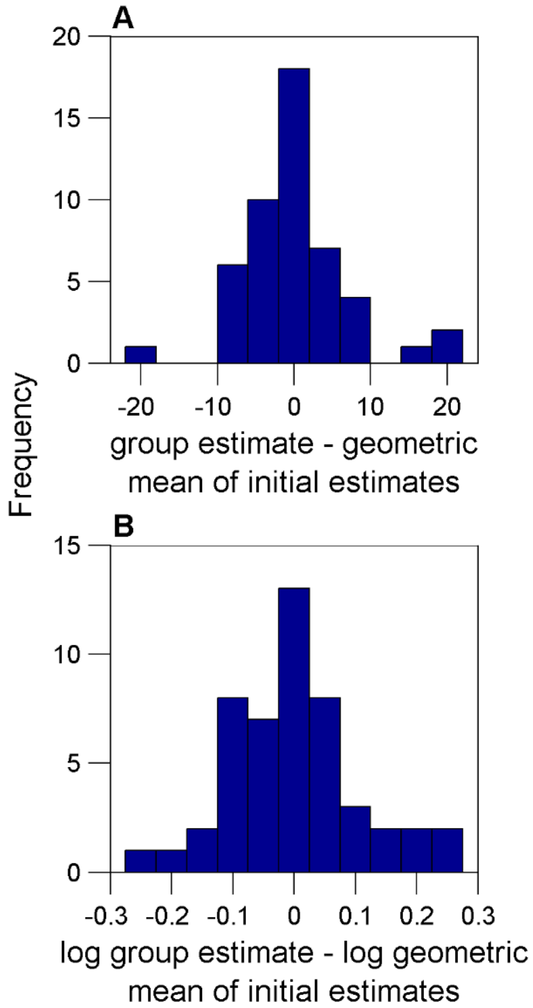

S13 Fig. The distribution of the difference between the group estimate and the mean of initial estimates. Shown are these variables unlogged (**A**) and logged (**B**). These distributions were used to determine whether the noise that occurs during group discussion relative to the geometric mean of the initial estimates is well approximated by a log-normal distribution (as used in the analysis described in the S1 Methods and corresponding to panel **B**) or a normal distribution (corresponding to panel **A**). Using Kolmogorov-Smirnov tests, both distributions were found to be not statistically different to a normal distribution ($p = 0.368$ for **A** and $p = 0.834$ for **B**). The difference between these two p values is 0.4655. To determine whether this difference could be expected by chance, we used a permutation test approach. Both distributions of differences were first standardised so that they are comparable, then we randomly shuffled whether each value from each of our test groups belonged to the

differences (panel **A** above) or the difference of the logarithms (**B**), and repeated the Kolmogorov-Smirnov tests as above on the randomised data set. This process was repeated 100,000 times, calculating the difference in the p values from the two Kolmogorov-Smirnov tests in each iteration. In only 4% (i.e. $p = 0.04$) of these random iterations was the p value for the difference in logarithms (**B**) minus the p value for the differences (**A**) greater than the equivalent statistic from the observed data (i.e. 0.4655). This suggests that the log-normal noise (**B**) is a statistically better fit than the non-logged normal noise (**A**).
