# Supplementary material for: Adolescents show collective intelligence which can be driven by a geometric mean rule of thumb
Source: PLoS One. 2018 Sep 24;13(9):e0204462. doi: 10.1371/journal.pone.0204462 (PMC6152954; doi:10.1371/journal.pone.0204462)
Supplement: S1 Table — (PDF) [file pone.0204462.s015.pdf]

S1 Table. Summary of statistical tests in Experiment 1. The LRT_{df} refers to the Likelihood Ratio Test with degrees of freedom. Random effects are either Group when multiple data points are associated with each group, or Group/Participant (i.e. participant identity is nested in group identity) when multiple data points are associated with each individual participant. In the Explanatory variable column, 'Stage' refers to Pre, Group or Post discussion estimates. 'Estimate type' refers to the observed groups' consensus estimates, the geometric mean of the individual initial estimates, the arithmetic mean of these estimates, or the arithmetic mean of the two initial estimates closest to one another before group discussion. 'Aggregation rule' refers to each of the different strategies for determining a group estimate (detailed in Fig 4). Significant effects at $p<0.05$ are marked in bold. Models are arranged in the order in which they are referred to in the main text.

Response variable and sample size (N)	Random intercept	Explanatory variable	LRT_{df}	p value
Absolute error (N = 441)	Group/ Participant	Age<br>Gender<br>Stage	$2.02_{1,433}$<br>$2.42_{1,433}$<br>**$50.66_{2,433}$**	0.16<br>0.12<br>**<0.001**
Probability of improving guess (N = 294)	Group/ Participant	Age<br>Gender<br>Stage	$0.56_{1,288}$<br>**$8.02_{1,288}$**<br>**$13.49_{1,288}$**	0.45<br>**0.0046**<br>**<0.001**
Absolute error in group estimate (N = 49)	NA	Mean age<br>Gender<br>Absolute error in mean × Range initial estimates	$3.015_{1,44}$<br>$0.42_{1,44}$<br>**$4.44_{1,43}$**	0.083<br>0.52<br>**0.035**
Absolute % change in group estimate vs. arithmetic mean (N = 49)	NA	Mean age<br>Gender<br>Range initial estimates	$2.68_{1,45}$<br>$0.016_{1,45}$<br>**$18.31_{1,45}$**	0.10<br>0.90<br>**<0.001**
Absolute % change in group estimate vs. geometric mean (N = 49)	NA	Mean age<br>Gender<br>Range initial estimates	$1.89_{1,45}$<br>$0.84_{1,45}$<br>$3.47_{1,45}$	0.17<br>0.36<br>0.062
Value of estimate (N = 196)	Group	Mean age<br>Gender<br>Range initial estimates × Estimate type	$0.018_{1,185}$<br>$0.30_{1,185}$<br>**$34.016_{3,184}$**	0.89<br>0.58<br>**<0.001**
Post-discussion estimate (N = 147)	Group	Age<br>Gender<br>Initial estimate	$2.10_{1,141}$<br>$0.014_{1,141}$<br>**$45.30_{1,141}$**	0.15<br>0.91<br>**<0.001**
Post-discussion estimate (N = 147)	Group	Age<br>Gender<br>log10 Initial estimate	$2.29_{1,141}$<br>$0.016_{1,141}$<br>**$54.90_{1,141}$**	0.13<br>0.90<br>**<0.001**
Absolute error in aggregation rule (N = 441)	Group	Mean age<br>Gender<br>Aggregation rule	$1.70_{1,428}$<br>$0.38_{1,428}$<br>**$67.88_{8,428}$**	0.19<br>0.54<br>**<0.001**
Absolute error in aggregation rule, low range groups (N = 315)	Group	Mean age<br>Gender<br>Aggregation rule	$0.99_{1,302}$<br>$0.56_{1,302}$<br>**$102.53_{8,302}$**	0.32<br>0.45<br>**<0.001**
Absolute error in aggregation rule, high range groups (N = 126)	Group	Mean age<br>Gender<br>Aggregation rule	$0.14_{1,113}$<br>$0.26_{1,113}$<br>**$88.38_{8,113}$**	0.70<br>0.61<br>**<0.001**
