# Supplementary material for: Adolescents show collective intelligence which can be driven by a geometric mean rule of thumb
Source: PLoS One. 2018 Sep 24;13(9):e0204462. doi: 10.1371/journal.pone.0204462 (PMC6152954; doi:10.1371/journal.pone.0204462)
Supplement: S2 Table — (PDF) [file pone.0204462.s016.pdf]

S2 Table. Absolute error of the geometric rule compared to the other aggregation rules and the observed consensus estimates (Experiment 1), for all groups, and then with the groups split by those with low (≤40) and high ranges (>40). Statistics are presented from the negative binomial GLMMs analysing error in group estimates as a function of gender, mean age and the aggregation rule applied to the initial estimates, with group as a random effect (S1 Table). Negative values in the Estimate column imply the geometric mean is outperformed by a particular rule (or the observed group estimates), while for positive values the converse is true. Significant differences ($p<0.05$) relative to the geometric mean are highlighted in bold. The notation for different rules is as in Fig 4.

Error in aggregation rule	Estimate	S.E.	z	p
All groups				
Observed	0.068	0.13	0.52	0.60
x_{13}	0.24	0.13	1.84	0.065
x_{23}	**0.26**	**0.13**	**2**	**0.046**
x_3	**0.74**	**0.13**	**5.8**	**<0.001**
x_1	**0.67**	**0.13**	**5.32**	**<0.001**
arit	0.090	0.13	0.7	0.48
x_2	0.11	0.13	0.87	0.39
x_{12}	**0.34**	**0.13**	**2.62**	**0.0088**
Groups with low ranges of initial estimates				
Observed	-0.018	0.094	-0.19	0.85
x_{13}	-0.032	0.094	-0.34	0.73
x_{23}	**-0.27**	**0.098**	**-2.75**	**0.0061**
x_3	**-0.20**	**0.099**	**-2.05**	**0.040**
x_1	**0.60**	**0.087**	**6.86**	**<0.001**
arit	-0.054	0.094	-0.57	0.57
x_2	0.013	0.093	0.14	0.89
x_{12}	**0.28**	**0.090**	**3.16**	**0.0016**
Groups with high ranges of initial estimates				
Observed	0.29	0.24	1.23	0.22
x_{13}	**0.95**	**0.23**	**4.15**	**<0.001**
x_{23}	**1.23**	**0.23**	**5.44**	**<0.001**
x_3	**1.97**	**0.22**	**8.76**	**<0.001**
x_1	**0.72**	**0.24**	**3.05**	**0.0023**
arit	**0.55**	**0.23**	**2.38**	**0.017**
x_2	0.34	0.24	1.44	0.15
x_{12}	0.38	0.24	1.62	0.11
