# Supplementary material for: Adolescents show collective intelligence which can be driven by a geometric mean rule of thumb
Source: PLoS One. 2018 Sep 24;13(9):e0204462. doi: 10.1371/journal.pone.0204462 (PMC6152954; doi:10.1371/journal.pone.0204462)
Supplement: S3 Table — (PDF) [file pone.0204462.s017.pdf]

S3 Table. Summary of statistical tests from Experiment 2. Random effects are either Group when multiple data points are associated with each group, or Group/Participant (i.e. participant identity is nested in group identity) when multiple data points are associated with each individual participant. 'Treatment' refers to the number of black versus total number of sweets in each jar, and 'Order' the testing order of each treatment for each group. Significant effects at p<0.05 are marked in bold. Models are arranged in the order in which they are referred to in the main text.

Response variable and sample size (N)	Random intercept	Explanatory variable	LRT_{df}	p value
Absolute error (N = 426)	Group/<br>Participant	Treatment<br>Initial or group estimate<br>Order	**20.46**$_{2, 417}$<br>**13.90**$_{1,417}$<br>**18.34**$_{2,417}$	**<0.001**<br>**<0.001**<br>**<0.001**
Absolute error in group estimate (N = 71)	Group	Treatment<br>Order<br>Absolute error in mean × Range initial estimates	1.79$_{2,62}$<br>0.38$_{2,62}$<br>**4.88**$_{1,61}$	0.41<br>0.83<br>**0.027**
Absolute % change in group estimate vs. arithmetic mean (N = 71)	Group	Treatment<br>Range initial estimates<br>Order	5.17$_{2,63}$<br>**14.32**$_{1,63}$<br>0.46$_{2,63}$	0.075<br>**<0.001**<br>0.80
Range initial estimates (N = 71)	Group	Order × Treatment	**10.73**$_{4,60}$	**0.030**
